# Deep Reinforcement Learning for Anti-Jamming Dynamic Spectrum Access: A Bootstrap Ensemble Approach with Echo State Network and Idle-Ratio Change Detection

**DOI:** 10.3390/s26154737

**Published:** 2026-07-26

**Authors:** Hao Jiang, Xin Bian, Mingqi Li

**Affiliations:** 1 Shanghai Advanced Research Institute, Chinese Academy of Sciences, Shanghai 201210, China; jianghao2022@shanghaitech.edu.cn (H.J.); bianx@sari.ac.cn (X.B.); 2 School of Information Science and Technology, Shanghaitech University, Shanghai 201210, China

**Keywords:** dynamic spectrum access, anti-jamming, deep reinforcement learning, echo state network, bootstrap ensemble, idle-ratio change detection

## Abstract

Dynamic spectrum access (DSA) is an effective technology to exploit spectrum for radio devices in complex electromagnetic environments. Systematic external jamming, such as swept and comb jamming, is a common form of jamming in anti-jamming communication scenarios. Deep reinforcement learning (DRL) is widely utilized to improve the performance of DSA. However, DRL-based DSA methods face challenges in generalizing across different jamming scenarios. In this paper, a DSA scheme based on a Bootstrap ensemble deep Q-network (BEDQN) integrated with an echo state network (ESN), termed ESN-BEDQN, is proposed to achieve fast and reliable access in scenarios where jamming patterns undergo sudden changes. The ESN provides low-complexity temporal memory to capture jamming patterns, while the BEDQN maintains multiple diverse readout heads to achieve fast exploration after ESN-BEDQN reset. Moreover, a jamming pattern change prediction method based on channel idle ratio detection using symmetric KL divergence is proposed to trigger network reset, i.e., Pred-Reset. Simulation results demonstrate that the ESN-BEDQN-based scheme achieves a near-zero collision rate under periodic jamming and recovers substantially faster than conventional deep Q-network (DQN)- and long short-term memory (LSTM)-DQN-based schemes in scenarios with abrupt jamming pattern changes. Furthermore, the Pred-Reset method can correctly capture jamming pattern changes and trigger network resets, achieving faster convergence than other baseline schemes across all tested scenarios.

## 1. Introduction

The rapid growth of wireless communication technologies and the proliferation of Internet of Things (IoT) devices have led to an unprecedented surge in spectrum demand [1]. Traditional static spectrum allocation policies assign frequency bands exclusively to licensed users, yet field measurements consistently reveal severe spectrum underutilization across time, frequency, and spatial domains [2]. This “spectrum scarcity paradox” has motivated the development of cognitive radio networks (CRNs) [3], in which secondary users (SUs) are allowed to opportunistically access temporarily idle licensed spectrum without causing harmful interference to primary users (PUs). Dynamic spectrum access (DSA), as the core enabling functionality of CRNs, significantly enhances spectrum efficiency by exploiting spectrum holes in real time.

Numerous approaches have been proposed for dynamic spectrum access (DSA), which can be broadly categorized into model-based and learning-based methods. Early studies primarily relied on model-based optimization techniques. Game-theoretic formulations [4,5] model spectrum access as a strategic interaction among competing users, where equilibrium solutions are derived under specific utility assumptions. Graph-based methods abstract interference constraints into graph coloring or graph segmentation problems [6]. Heuristic strategies [7] offer computationally efficient solutions but require careful handcrafted design tailored to specific scenarios. To overcome the modeling limitations of these approaches, deep reinforcement learning (DRL) has been introduced to DSA and extensively investigated, including applications to dynamic spectrum sensing and aggregation [8], simultaneous sensing and channel access [9], listen-after-collision mechanisms with improved exploration [10], distributed multi-agent spectrum access with partial observations [11], and actor–critic architectures for dynamic multichannel access [12]. Recent studies have further extended multi-agent DRL to more complex distributed environments, including satellite–terrestrial integrated networks [13,14] and emergency cognitive radio systems [15]. In addition, DRL has also been applied to optimize age of information (AoI) in ambient backscatter-assisted energy harvesting cognitive radio networks with cooperative spectrum sensing, highlighting the importance of freshness-aware and energy-efficient spectrum access [16]. Beyond conventional DRL frameworks, recent studies have explored more advanced learning paradigms, such as federated and online learning, to enable distributed spectrum access while preserving data privacy and adapting to user mobility [17]. Despite these advances, most existing DSA studies primarily focus on spectrum efficiency and resource allocation, and only limited attention has been paid to scenarios involving external jamming, especially when the jamming pattern changes abruptly.

In practice, however, the electromagnetic environment can be highly dynamic. The DARPA Spectrum Collaboration Challenge (SC2) [18,19] underscored the critical need for wireless systems to autonomously adapt to unpredictable spectrum variations in real time. While SC2 focused primarily on collaborative spectrum sharing among autonomous radios, the broader lesson—that adaptation speed and autonomy are essential when spectrum conditions change abruptly—applies equally to scenarios involving external interference. This paper concentrates on a particularly challenging yet practical subset of such scenarios: non-reactive jamming with abrupt pattern switching. In this setting, the jammer does not intelligently track or react to the SU’s access behavior; rather, its interference pattern (e.g., periodic, sweeping, or random) is pre-scripted but can switch unpredictably among several modes. Such non-reactive jamming with abrupt pattern switches is common in practice, arising from sources such as industrial unintentional interferers, low-cost periodic jammers, or pre-programmed electronic countermeasures. When the jamming pattern changes, the statistical properties of spectrum holes shift abruptly, causing previously learned access policies to suffer severe performance degradation or even fail entirely. This calls for anti-jamming DSA solutions that can rapidly recover performance after pattern switches, without requiring prior knowledge of when a switch occurs.

Even when DRL is adopted as the underlying learning framework for this problem, standard methods face two fundamental challenges in such abruptly changing environments. First, fully connected networks process sensing observations independently and lack explicit modeling of temporal dependencies in spectrum occupancy patterns. Although recurrent architectures such as long short-term memory (LSTM) can capture historical dependencies, they incur heavy online training overhead and slow convergence [20,21]. Second, DRL relies on experience replay and a decaying ε-greedy exploration mechanism. After ε decays to its final value, exploration efficiency drops substantially; when an abrupt environmental change occurs, the replay buffer becomes flooded with obsolete transitions that severely mislead policy updates, and the agent struggles to re-explore autonomously [20,22].

A growing body of work has begun to consider time-varying and non-stationary environments in DSA [12,22,23,24]. Sheng et al. [23] proposed a transfer RL algorithm to improve the effectiveness of DRL models in dynamic spectrum environments. However, this work focused mainly on single SU scenarios and requires prior knowledge of the change point. Wang et al. [22] designed an adaptive mechanism to detect environmental changes and retrain the model when the accumulated reward decreased by a given threshold. Similarly, retraining in response to environmental changes was also discussed in Refs. [12,24]. Some studies have also explored reward-decreasing response strategies to handle time-varying environments online, but they remain constrained by the low exploration efficiency of ε-greedy after annealing [22]. Overall, while these methods provide valuable insights into handling environmental dynamics, they either require prior knowledge of the change point, rely on reactive performance monitoring, or are limited by the exploration bottleneck of ε-greedy, making them insufficient for fast and autonomous adaptation to abrupt jamming changes.

To enable fast and robust adaptation under abrupt environmental changes, two key challenges must be addressed: lightweight temporal modeling and sustained exploration. For temporal modeling, while LSTM is the most widely adopted recurrent architecture in DRL-based DSA, it requires gradient-based training of the recurrent weights, incurring high online computational cost and slow adaptation when the jamming pattern changes. The echo state network (ESN) [25] operates with fixed random recurrent connections and trains only the readout layer, substantially reducing online computation and preserving temporal memory without weight update delays—an advantage that has been empirically validated in DSA settings [26,27]. For exploration, methods such as NoisyNet and parameter-space noise can sustain exploration without explicit ε-greedy decay, but they typically increase architectural complexity or require careful hyperparameter tuning. The bootstrap ensemble [28] achieves deep exploration through lightweight, randomized value estimation across multiple heads, naturally integrating with the ESN’s multi-readout structure and adding minimal computational overhead. For change detection, alternative approaches often rely on cumulative reward monitoring [22] or fixed-threshold schemes; these are either reactive, requiring multiple steps to confirm a change, or demand prior knowledge of the environment. We instead design Pred-Reset to monitor the symmetric KL divergence of channel idle ratios, directly capturing statistical shifts in spectrum occupancy and enabling rapid, unsupervised reset triggering without any change-point prior.

Focusing on DSA in time-varying, non-reactive jamming environments, this paper proposes a coordinated ESN-BEDQN framework that explicitly couples temporal modeling, exploration, and environment adaptation into a unified learning framework. Specifically, ESN provides low-complexity temporal representation, the bootstrap ensemble enables annealing-free exploration, and an idle-ratio-based change detection mechanism ensures timely adaptation to environmental changes without manual threshold setting or prior knowledge of change points. The main contributions are as follows:

1. Low-complexity temporal memory: An echo state network serves as the Q-network backbone, exploiting the inherent echo state property of the reservoir to capture temporal regularities in jamming patterns. Since only the readout weights require training, the online computational cost is substantially lower than that of an LSTM of comparable scale.
2. High-efficiency Bootstrap ensemble exploration: Multiple diversified readout heads are maintained [28]. Through random head selection, the ensemble achieves higher exploration efficiency than conventional ε-greedy strategies after reset, accelerating policy convergence.
3. Sensitive adaptive reset: An adaptive reset mechanism, termed Pred-Reset, is designed based on symmetric KL divergence change detection of channel idle ratios. Upon detecting an environmental change, the experience replay buffer is immediately cleared, and the reservoir state is reset, enabling the agent to rapidly discard obsolete knowledge.
4. Intrinsic reward design: An intrinsic reward based on channel prediction error is optionally introduced to provide additional exploration drive when jamming patterns change abruptly.

In summary, existing studies either focus on improving spectrum access efficiency using DRL or address environmental dynamics through reactive adaptation mechanisms. However, few works jointly consider lightweight temporal modeling, sustained exploration, and rapid adaptation under abruptly changing jamming environments. Simulation results under both static periodic jamming and time-varying composite jamming scenarios demonstrate that ESN-BEDQN significantly outperforms conventional DQN and LSTM-DQN baselines in convergence speed, steady-state success access rate, and resilience to pattern changes, achieving a recovery speed substantially faster than the compared schemes. Notably, temporal memory alone suffices for smooth adaptation when jamming structures remain similar, whereas hard reset becomes essential when the jamming structure changes fundamentally.

The remainder of this paper is organized as follows. Section 2 presents the system model and problem formulation. Section 3 details the design of the ESN-BEDQN algorithm. Section 4 reports the simulation results and comparative analysis. Section 5 concludes the paper.

## 2. System Model and Problem Formulation

### 2.1. Network and Signal Model

**Figure 1:**
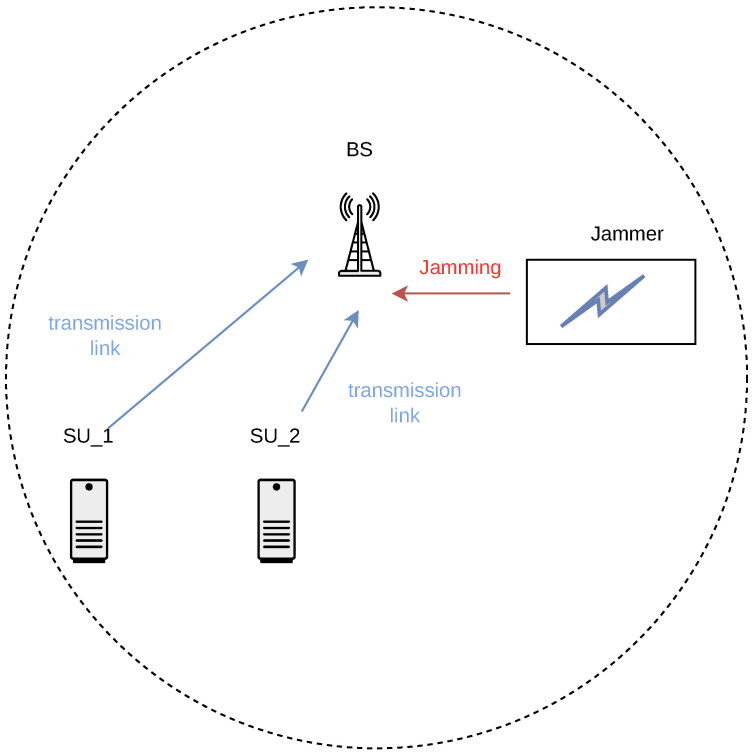
Example of system model.

Consider a communication scenario deployed within a 5 km × 5 km region, consisting of secondary user (SU) transmitter–receiver pair and independent jammers, As illustrated in Figure 1. The licensed spectrum is divided into *K* orthogonal channels, each with bandwidth *B*, which are non-overlapping in the frequency domain. The SU aims to opportunistically access and transmit on channels that are temporarily free from jamming. The jammer locations and transmission strategies are unknown to the SU.

System time is divided into equal-duration slots indexed by t=1,2,… Each slot consists of a spectrum sensing phase and a data transmission phase. During the sensing phase, the SU transmitter performs wideband spectrum sensing across all *K* channels. To focus on MAC-layer access decisions, sensing is assumed to be ideal, i.e., sensing errors and receiver noise are neglected. This assumption is adopted to isolate the impact of abrupt jamming pattern changes, which already pose significant challenges to DRL-based approaches even in the absence of sensing uncertainty. In addition, it is reasonable in scenarios with high SNR, sufficient sensing duration, or cooperative sensing, where sensing errors are negligible. In practice, imperfect sensing (e.g., missed detections and false alarms) would affect the ACK-derived idle-ratio estimates used by Pred-Reset, and extending the proposed framework to such cases is left for future work. The sensing outcome is represented by a binary vector:

(1) $$s_{t}^{\mathrm{sense}}=\left[s_{t,1}^{\mathrm{sense}},s_{t,2}^{\mathrm{sense}},\ldots ,s_{t,K}^{\mathrm{sense}}\right],$$

where $s_{t,k}^{\mathrm{sense}}\in \left\{0,1\right\}$, with 1 indicating that channel *k* is sensed as idle and 0 indicating busy (jammed). During the data transmission phase, the SU selects a channel $a_{t}\in \left\{1,2,\ldots ,K\right\}$ for access. If no channel is deemed usable, the SU may choose the idle action $a_{t}=a_{\mathrm{idle}}$.

Both path loss and Rayleigh fading [29] are considered. The channel gain from the SU transmitter to the SU receiver on channel *k* is

(2) $$h_{t,k}^{\mathrm{SU}}=g_{\mathrm{SU}}\cdot d_{\mathrm{SU}}^{-\alpha }\cdot {\left|{\tilde{h}}_{t,k}^{\mathrm{SU}}\right|}^{2},$$

where $g_{\mathrm{SU}}$ is a constant incorporating antenna gains and carrier frequency, $d_{\mathrm{SU}}$ is the distance between the SU transmitter and the receiver, α is the path-loss exponent, and ${\tilde{h}}_{t,k}^{\mathrm{SU}}∼\mathrm{CN}\left(0,1\right)$ is the unit-power Rayleigh fading coefficient, independent and identically distributed across slots and channels. The channel gain from each jammer to the SU receiver follows the same expression.

Let $P_{\mathrm{SU}}$ denote the SU transmit power, $P_{J}$ the jammer transmit power, and $\sigma^{2}$ the additive white Gaussian noise power. When the SU accesses channel *k*, the SINR at the SU receiver is

(3) $${\mathrm{SIN}R}_{t,k}=\frac{P_{\mathrm{SU}}\cdot h_{t,k}^{\mathrm{SU}}}{\sigma^{2}+P_{J}\cdot h_{t,k}^{J}\cdot j_{t,k}},$$

where $j_{t,k}\in \left\{0,1\right\}$ is the jamming indicator: $j_{t,k}=1$ means channel *k* is jammed at slot *t*, and $j_{t,k}=0$ means it is not. Let $\gamma_{\mathrm{th}}$ denote the SINR threshold for successful demodulation. Transmission is successful if and only if $a_{t}=k$ and ${\mathrm{SIN}R}_{t,k}\geq \gamma_{\mathrm{th}}$. If the SU chooses the idle action, no transmission occurs.

At the end of each slot, the SU receiver feeds back a binary acknowledgment signal ${\mathrm{ACK}}_{t}\in \left\{0,1\right\}$ to the transmitter, where 1 indicates success and 0 indicates failure. The SU can use this signal to update its estimate of the state of each channel. The time-varying characteristics of the jamming indicator $j_{t,k}$, which dominate the dynamics of spectrum holes, are modeled in detail in Section 2.2.

### 2.2. Time-Varying Jamming Model

This work considers two elementary jamming signal types: comb jamming and sweeping jamming. The jammers construct time-varying attack strategies by dynamically combining and switching between these two signals.

Comb jamming: The jammer simultaneously attacks a fixed subset of channels $K_{\mathrm{comb}}\subset \left\{1,2,\ldots ,K\right\}$ in each slot. This subset remains unchanged within a given mode:

(4) $$j_{t,k}=\left\{\begin{array}{cc} 1, & \mathrm{if}\;k\in K_{\mathrm{comb}},\\ 0, & \mathrm{otherwise}. \end{array}\right.$$

Sweeping jamming: The jammer attacks only one channel per slot, sweeping in a fixed direction. Two basic sweeping directions are considered: ascending (from CH 0 to CH 7) and descending (from CH 7 to CH 0). The basic sweeping model can be extended to various variants by adjusting the sweeping width and dwell time. For example, concatenating descending and ascending segments produces more complex sweeping patterns. The sweeping modes used in the simulations are specific instances of these variants. The sweeping jamming indicator is $j_{t,k}=1$ if $k=c_{t}$, and 0 otherwise, where $c_{t}$ is the index of the channel attacked at slot *t*.

Time-varying hybrid patterns: The jammers combine or cycle the two elementary signals in different ways across stages. The environment switches among several predefined hybrid patterns, each specifying the sequential arrangement or cycling rule of the two signals during that stage. Let $m_{t}\in \left\{1,2,\ldots ,M\right\}$ denote the hybrid pattern index at slot *t*. The sequence $\left\{m_{t}\right\}$ is piecewise stationary: the environment remains in a stationary regime $E_{i}$ with hybrid pattern $m^{\left(i\right)}$ for a duration of $T_{\mathrm{env}}$ slots, then transitions to the next pattern $m^{\left(i+1\right)}$. The regime duration and transition times are completely unknown to the SU.

### 2.3. Problem Formulation

This work adopts a half-duplex cognitive radio scheme: the SU performs wideband spectrum sensing at the beginning of each slot, then selects a channel for access and completes data transmission without further sensing during transmission. This scheme requires no full-duplex hardware support, avoiding the high cost and self-interference cancelation challenges associated with full-duplex radios, and is commonly adopted in practical cognitive radio systems. Under this scheme, the action, state, and reward of the reinforcement learning formulation are defined as follows.

State: The state at slot *t* is directly taken as the binary sensing vector $o_{t}=s_{t}^{\mathrm{sense}}$, with dimensionality *K*. This state contains only the current sensing snapshot without any manually designed features. Although no explicit history is included, the echo state network reservoir introduced in Section 3 possesses inherent short-term memory and can automatically extract temporal jamming patterns from the sequentially input observations, thereby eliminating the need to incorporate a history window into the state.

Action: The SU selects one channel for access or chooses not to access in each slot. The action space is $A=\left\{0,1,\ldots ,K-1\right\}\cup \left\{a_{\mathrm{idle}}\right\}$, where $a_{t}=k$ denotes accessing channel *k*, and $a_{\mathrm{idle}}$ denotes remaining silent.

Reward: The reward is a composite of an extrinsic reward and an intrinsic reward: $R_{t}=r_{t}^{\mathrm{ext}}+\lambda r_{t}^{\mathrm{int}}$, where λ≥0 is the intrinsic reward weight. The extrinsic reward is determined by the transmission outcome: +1 for success, −1 for failure, and 0 for idle. The symmetric weights +1 and −1 drive the SU to actively utilize idle spectrum while avoiding jammed channels. Since transmission failure occurs only when the selected channel is under jamming, no distinction in penalty values is necessary. The intrinsic reward is defined as the prediction error of the selected channel: $r_{t}^{\mathrm{int}}=e_{a_{t}}^{\mathrm{pred}}$, where $e_{k}^{\mathrm{pred}}$ is a channel-wise prediction error maintained internally (detailed in Section 3.5). In the experiments, λ=0.1 is adopted, while the intrinsic reward mechanism remains available as an extension for scenarios with more frequent or severe jamming pattern changes.

Objective: The SU aims to learn a policy π(a∣o) that maximizes the expected cumulative discounted reward:

(5) $$J\left(\pi \right)=E_{\pi }\left[\sum_{\tau =0}^{\infty }\gamma^{\tau }R_{t+\tau }\right],$$

in time-varying jamming environments, where γ∈(0,1) is the discount factor. The core challenge of this MDP lies in the fact that the time-varying nature of jamming patterns causes abrupt changes in environmental dynamics, requiring the algorithm to simultaneously possess temporal memory, change awareness, and policy reset capabilities. This is precisely the motivation for the algorithm design presented in Section 3.

## 3. ESN-Based Bootstrap Ensemble Deep Q-Network (ESN-BEDQN)

To address the MDP with abrupt jamming pattern changes formulated in Section 2, this section proposes ESN-BEDQN, a Bootstrap ensemble deep Q-network algorithm integrating an echo state network with idle-ratio change detection. The algorithm tackles the challenges posed by abrupt jamming pattern changes through three synergistic modules: an ESN reservoir providing low-cost temporal memory, a Bootstrap ensemble delivering higher exploration efficiency than ε-greedy after reset, and an adaptive reset mechanism based on KL divergence change detection of channel idle ratios for rapid perception of and response to environmental changes.

### 3.1. Limitations of Standard DQN

Standard DQN employs a fully connected neural network as the Q-function approximator and relies on an experience replay buffer to break temporal correlations among samples. However, in the time-varying jamming environment described in Section 2.2, this architecture faces two fundamental problems.

First, fully connected networks lack the ability to explicitly model temporal dependencies. Although concatenating the sensing results of several past slots can partially compensate, such fixed windows cannot adequately capture long-range temporal regularities, and the choice of window length depends on prior knowledge of the jamming pattern. Long short-term memory (LSTM) networks can capture historical dependencies, but they incur heavy online training overhead and slow convergence.

Second, DQN adopts an ε-greedy exploration strategy, with ε typically decaying from an initial high value to a final value (e.g., 0.01). After ε decays to its final value, exploration efficiency drops substantially. When an abrupt environmental change occurs, the replay buffer becomes flooded with obsolete transitions that severely mislead policy updates, and the extremely low exploration rate prevents the agent from autonomously escaping the obsolete policy.

### 3.2. ESN-Q Network: Low-Cost Temporal Memory

To equip the agent with temporal modeling capability while avoiding the online training overhead of LSTM, this work adopts an echo state network as the backbone of the Q-network. The core of ESN is a large, sparsely connected, randomly initialized recurrent neural network (the reservoir). The internal connection weights of the reservoir are randomly generated and then kept fixed; only the readout weights from the reservoir state to the output need to be trained.

Network structure: The ESN-Q network consists of three layers. The input layer receives the state vector $o_{t}$ (dimension *K*) defined in Section 2.3, which is mapped via random input weights $W_{\mathrm{in}}$ and injected into the reservoir. The reservoir contains $N_{\mathrm{res}}=64$ neurons, and the spectral radius of its internal connection matrix $W_{\mathrm{res}}$ is set to ρ=0.8 to guarantee the echo state property—the reservoir possesses fading memory of recent inputs while automatically forgetting distant history. The reservoir state update equation is

(6) $$h_{t}=\tanh\left(W_{\mathrm{in}}o_{t}+W_{\mathrm{res}}h_{t-1}\right).$$

To enhance information flow, the actual extended state vector used is the concatenation of the reservoir state and the scaled original input:

(7) $$x_{t}=\left[h_{t};u_{t}\right],$$

where $u_{t}$ is the vector obtained after applying input scaling and shifting to $o_{t}$. This concatenation enables the readout layer to simultaneously exploit the temporal features encoded by the reservoir and the instantaneous information of the current input.

Readout and training: The output layer computes the Q-value for each action *a* as

(8) $$Q\left(o_{t},a\right)=W_{\mathrm{out}}^{\left(a\right)}\cdot x_{t},$$

where $W_{\mathrm{out}}^{\left(a\right)}$ is the readout weight vector corresponding to action *a*. During training, only $W_{\mathrm{out}}$ needs to be updated via stochastic gradient descent, with computational complexity substantially lower than the backpropagation overhead of an LSTM of comparable scale. When the environment changes, only a few new samples are required to quickly adjust the readout layer without retraining the entire network. Meanwhile, the fixed random projection of the reservoir naturally acts as a regularizer, effectively mitigating overfitting under small sample sizes.

Unlike LSTM/GRU-based architectures, ESN separates representation learning and readout training, avoiding backpropagation through time and enabling fast adaptation under abrupt environmental changes. Moreover, the fixed random reservoir acts as a universal nonlinear feature mapper, providing stable representations under abrupt jamming pattern changes and limited data conditions.

### 3.3. Bootstrap Ensemble: High-Efficiency Sustained Exploration

To maintain sufficient exploration capability when jamming patterns change abruptly, this work introduces a Bootstrap ensemble method as an alternative to the conventional ε-greedy strategy. The algorithm maintains $N_{\mathrm{ensemble}}=5$ independent readout weight matrices $W_{\mathrm{out}}^{\left(1\right)},\ldots ,W_{\mathrm{out}}^{\left(N_{\mathrm{ensemble}}\right)}$, each corresponding to an ensemble head. All heads share the same reservoir and input weights; only the readout weights are independent.

Decision-making: At each slot, the algorithm uniformly selects one head *k* from the $N_{\mathrm{ensemble}}$ heads and greedily chooses an action based on the Q-values of that head:

(9) $$a_{t}=\arg\max_{a}Q_{k}\left(o_{t},a\right).$$

Since the readout weights of different heads differ from one another due to differentiated sampling of training data, this random head selection naturally sustains exploration without any annealing schedule. In evaluation mode, the average Q-value over all heads is used as the final Q-value to eliminate randomness.

Training: The training of the Bootstrap ensemble relies on differentiated sampling. For each head *k*, before each mini-batch update, an independent Bernoulli mask $m_{k}\in {\left\{0,1\right\}}^{\mathrm{batch\_size}}$ is generated, where each sample is selected to participate in the weight update of that head with probability $p_{\mathrm{mask}}=0.6$. This is equivalent to constructing a different training data subset for each head, forcing them to learn different Q-value approximations. During stationary periods, the heads tend to agree, and randomly selecting any head yields good actions; after an environmental change, the heads adapt to the new environment at different speeds due to their distinct training data, and the resulting prediction disagreement promotes sufficient re-exploration.

Target network: Each ensemble head maintains a target readout weight $W_{\mathrm{out}}^{(k)\mathrm{target}}$, which is updated with the current weights every C=10 learning steps. The target Q-value is computed using the target weight of the *k*-th head rather than the average over all heads, to preserve the independence of each head:

(10) $$y_{i}=r_{i}+\gamma \cdot \max_{a}Q_{k}^{\mathrm{target}}\left(o_{i+1},a\right).$$

This mechanism replaces the conventional ϵ-greedy exploration strategy and enables sustained exploration without an explicit decay schedule. The bootstrap mechanism induces functional diversity among Q-value estimators, leading to implicit uncertainty-driven exploration through stochastic head selection.

### 3.4. Environmental Change Detection and Adaptive Reset

The ESN and Bootstrap ensemble described above address the problems of temporal modeling and exploration during stationary periods, but obsolete knowledge must still be rapidly discarded when the environment changes abruptly. This work designs an adaptive reset mechanism termed Pred-Reset, based on KL divergence change detection of channel idle ratios.

Idle-ratio estimation: Each channel *k* maintains a sliding window of length W=200, recording the true idle state (obtained via ACK feedback) of that channel over the most recent *W* slots. The idle ratio is defined as the proportion of idle slots within the window:

(11) $$p_{t,k}^{\mathrm{idle}}=\frac{1}{W}\sum_{i=0}^{W-1}\left(1-j_{t-i,k}\right).$$

This estimate reflects the recent proportion of time each channel is free from jamming and serves as a compact representation of the spectrum occupancy statistics. The idle ratio is chosen as the detection signal because it directly captures channel variations in spectrum occupancy: when the jamming pattern changes, the idle-ratio distribution shifts immediately, providing a more responsive trigger than reward-based signals that require averaging over multiple decision steps.

Change detection: The symmetric KL divergence between the current idle-ratio vector and that of 30 steps ago is computed in real time as the change metric:

(12) $$D_{t}=D_{\mathrm{KL}}\left(p_{t}^{\mathrm{idle}}∥p_{t-W}^{\mathrm{idle}}\right)+D_{\mathrm{KL}}\left(p_{t-W}^{\mathrm{idle}}∥p_{t}^{\mathrm{idle}}\right).$$

The exponentially weighted moving mean $\mu_{D}$ and standard deviation $\sigma_{D}$ of $D_{t}$ are maintained (update rate α=0.01). An environmental change is declared when $D_{t}>\mu_{D}+\beta \cdot \sigma_{D}$, where the sensitivity coefficient β=2.5. The symmetric KL divergence is adopted here because it quantifies the dissimilarity between two idle-ratio distributions without assuming any specific parametric form. Compared to simpler metrics such as mean absolute difference, KL divergence is more sensitive to changes in the overall distribution shape, enabling reliable detection even when the average idle ratio changes only modestly but the per-channel distribution shifts significantly. The symmetric form avoids the directional bias of one-sided KL divergence, ensuring that any structural change in the idle-ratio distribution is detected regardless of its direction. The idle ratio directly captures channel variations in spectrum occupancy, providing a more immediate reflection of environmental changes than reward-based signals, which require averaging over multiple decision steps to reveal performance degradation.

Adaptive reset strategy: Upon detecting a change, hard reset operations are executed: the experience replay buffer is cleared, discarding all historical experience; the reservoir internal state is reset to the zero vector; and half of the Bootstrap ensemble head readout weights are randomly reinitialized to increase exploration diversity. After triggering, a cooldown period of 500 steps is entered, during which no new detection signals are responded to, preventing consecutive repeated triggers.

The reset mechanism is designed as a lightweight statistical change detector based on idle-ratio distribution shifts, enabling fast adaptation to abrupt jamming pattern changes without relying on delayed reward signals.

### 3.5. Intrinsic Reward Design

To further enhance the exploration drive when jamming pattern changes are more frequent or severe, the algorithm optionally enables an intrinsic reward based on channel prediction error. For each channel *k*, a lightweight exponential-smoothing predictor is maintained: the true idle state of the previous slot $s_{t-1,k}^{\mathrm{true}}$ serves as the prediction ${\hat{s}}_{t,k}^{\mathrm{true}}$ for the current slot. The one-step error $|{\hat{s}}_{t,k}^{\mathrm{true}}-s_{t,k}^{\mathrm{true}}|$ is exponentially smoothed with a factor of 0.1 to obtain $e_{k}^{\mathrm{pred}}$. The intrinsic reward is then $r_{t}^{\mathrm{int}}=e_{a_{t}}^{\mathrm{pred}}$, encouraging exploration of channels with high uncertainty and temporal variability, which is particularly beneficial after abrupt environmental changes.

### 3.6. Algorithm Flow

The complete training procedure of ESN-BEDQN is summarized in Algorithm 1.

**Algorithm 1:** ESN-BEDQN Training Procedure      **Input:** *K* channels, $N_{\mathrm{ensemble}}=5$, $N_{\mathrm{res}}=64$, buffer M=290      **Output:** Trained readout weights $W_{\mathrm{out}}^{\left(k\right)}$, $k=1,\ldots ,N_{\mathrm{ensemble}}$      **Initialize:**      1       Randomly generate and fix $W_{\mathrm{in}}$, $W_{\mathrm{res}}$ (ρ=0.8)      2       Initialize $W_{\mathrm{out}}^{\left(k\right)}$ to small random values      3       Set target $W_{\mathrm{out}}^{(k)\mathrm{target}}=W_{\mathrm{out}}^{\left(k\right)}$      4       Initialize D=∅, h=0, idle-ratio windows (W=200)      **Training loop:**      5       **for** t=1,2,… 
**do**      6            Perform wideband sensing, obtain $o_{t}$      7            Randomly select head $k∼\mathrm{Uniform}\{1,\ldots ,N_{\mathrm{ensemble}}\}$      8            Compute $x_{t}=\left[h_{t};u_{t}\right]$      9            Select $a_{t}=\arg\max_{a}W_{\mathrm{out}}^{\left(k\right)}\cdot x_{t}$      10          Execute $a_{t}$, receive $R_{t}$, ACK, $o_{t+1}$      11          $h_{t+1}=\tanh\left(W_{\mathrm{in}}u_{t+1}+W_{\mathrm{res}}h_{t}\right)$      12          Store $\left(x_{t},a_{t},R_{t},x_{t+1}\right)$ in D      13          Update $p_{t,k}^{\mathrm{idle}}$ for all channels      14          Compute $D_{t}$, update $\mu_{D}$ and $\sigma_{D}$      15          **if** $D_{t}>\mu_{D}+\beta \sigma_{D}$ and cooldown elapsed **then**      16              Clear D, reset h=0      17              Randomly reinitialize half of $W_{\mathrm{out}}^{\left(k\right)}$      18              Enter cooldown period (500 steps)      19          **end if**      20          **if** |D|≥batch_size **then**      21              **for** each head $k=1,\ldots ,N_{\mathrm{ensemble}}$ **do**      22                  Generate Bernoulli mask $m_{k}$ ($p_{\mathrm{mask}}=0.6$)      23                  Compute TD error, update $W_{\mathrm{out}}^{\left(k\right)}$      24              **end for**      25              Every C=10 steps: $W_{\mathrm{out}}^{(k)\mathrm{target}}=W_{\mathrm{out}}^{\left(k\right)}$      26          **end if**      27     **end for**

## 4. Simulation Results and Analysis

This section evaluates the performance of the proposed ESN-BEDQN algorithm through three simulation experiments. Section 4.1 presents the global simulation setup and common hyperparameters. Section 4.2 compares DQN-ESN with DQN-MLP under static periodic jamming to verify the necessity of temporal memory. Section 4.3 investigates the rapid adaptation capability under jamming pattern changes through a unified comparison of six schemes. Section 4.4 verifies the effectiveness of Pred-Reset online detection as a replacement for the ideal reset signal.

### 4.1. Simulation Setup

All experiments are implemented in Python 3.7 based on NumPy 1.21.6. Consider a communication scenario within a 5 km × 5 km region, where a secondary user (SU) transmitter–receiver pair operates in the presence of two independent jammers. The spectrum is divided into *K* orthogonal channels, and the SU has a transmit power of 20 mW, and the jammer operates at 40 mW. The channel follows a frequency-flat Rayleigh fading model. To focus on MAC-layer access decisions, physical-layer details such as sensing errors, low-SNR degradation, and adjacent-channel interference are neglected. In each slot, the SU performs wideband spectrum sensing and selects one channel for access, or remains silent if no channel is chosen. The performance metrics are the average success access rate and the average collision rate, both evaluated every 200 steps. The policy recovery steps after a change, defined as the number of steps required to regain 90% of the steady-state performance in the new environment, are reported in tables.

Table 1 summarizes the global hyperparameters. The algorithms are DQN-MLP (standard DQN with a two-layer fully connected network, hidden layer sizes 128 and 64, ReLU activation, trained via SGD, ε-greedy exploration), used in Section 4.2; DQN-ESN (ESN replaces the fully connected network, 64 reservoir neurons, spectral radius 0.8, input scaling 2.0, input shift −1.0, only the readout weights are trainable, trained via SGD, ε-greedy exploration), used in Section 4.2 and Section 4.3; DQN-LSTM (one-layer LSTM with 64 hidden units followed by a fully connected output layer, trained via SGD, ε-greedy exploration), used in Section 4.3; ESN-BEDQN (proposed, ESN + Bootstrap ensemble + Pred-Reset), used in Section 4.3 and Section 4.4. In the ablation experiments of Section 4.3, DQN-ESN, DQN-LSTM, and ESN-BEDQN are each additionally configured with a no-reset version (w/o reset), in which the idle-ratio change detection, experience buffer clearing, and reservoir state reset are disabled, while all other structures remain identical. In the figures, “Reset” denotes schemes with reset enabled, and “NoSet” denotes schemes without reset. All algorithms share the same learning rate, discount factor, replay buffer capacity, and batch size. All algorithms use stochastic gradient descent (SGD) as the optimizer. ε decays from 1.0 to 0.01 over 20,000 steps. For each configuration, multiple runs with different random seeds were conducted. The results shown in the figures correspond to representative runs, as the performance trends are consistent across different seeds.

### 4.2. Static Periodic Jamming

This section verifies the necessity of temporal memory under static periodic jamming. The jammer applies synchronous pulsed jamming to all channels: T=3 for the first 20,000 steps, then T=5 for the remaining 20,000 steps. K=4, single SU. DQN-ESN is compared with DQN-MLP. Both use ε-greedy (ε: 1.0 → 0.01, 20,000 steps), trained for 40,000 steps, evaluated every 100 steps.

**Figure 2:**
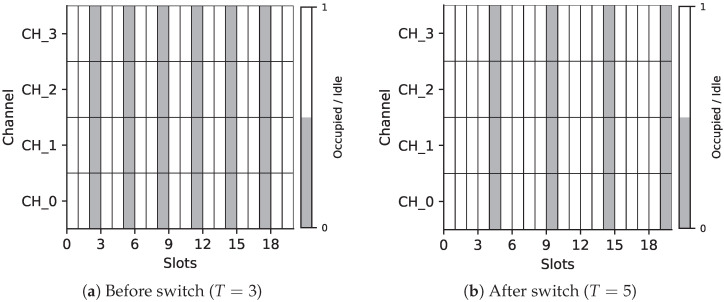
Spectrum occupancy grid of the static periodic jamming scenario (K=4). White = idle, dark = jammed.

Figure 2 shows the spectrum occupancy grid around the switch.

**Figure 3:**
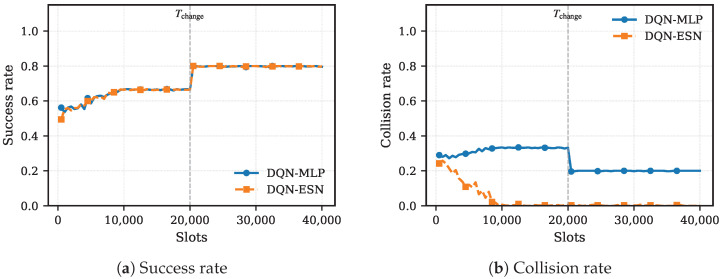
Success access rate and collision rate curves of DQN-ESN and DQN-MLP under static periodic jamming (K=4). The gray dashed line marks the change point.

Figure 3 plots the success and collision curves. DQN-ESN converges to a near-zero collision rate within 100 steps, achieving the theoretical maximum success rate determined by the proportion of non-jammed slots. For period T=3, one out of every three slots is jammed, so the maximum achievable success rate is (T−1)/T=2/3≈66.7%. For T=5, the maximum success rate is 4/5=80.0%. After the switch, its collision rate briefly spikes then returns to near zero. In contrast, DQN-MLP achieves the same success rates but with collision rates of 33.3% (T=3) and 20.0% (T=5), exactly matching the proportion of jamming slots (Table 2). This indicates that DQN-MLP has learned a memoryless “always access” policy, colliding with the jammer in every jamming slot.

These results confirm that temporal memory is indispensable in DSA. ESN extracts temporal structure without backpropagation training of recurrent weights, providing a lightweight foundation for the subsequent scenarios with abrupt jamming pattern changes.

### 4.3. Rapid Adaptation Under Jamming Pattern Changes

This section evaluates the rapid adaptation capability under time-varying composite jamming. Two jammers operate in either superposition mode (both active) or alternating mode (one active per slot). Four scenarios are formed: Scenario 1 (alternating → superposition, different-mode switch); Scenario 2 (superposition → alternating, different-mode switch); Scenario 3 (alternating A → alternating B, same-mode parameter switch); Scenario 4 (superposition A → superposition B, same-mode parameter switch). K=8, single SU, 40,000 training steps per scenario, change at step 20,001...Scenario 4 (superposition A → superposition B, same-mode parameter switch). K=8, single SU, 40,000 training steps per scenario, change at step 20,001.

The jamming patterns considered in this section are designed to reflect practical interference scenarios encountered in communication applications, where multiple jammers may operate in superposition or alternating modes. By composing comb and sweeping jamming signals into structured mode-switching sequences, we aim to systematically evaluate the algorithm’s adaptation capability to abrupt environmental changes. The controlled variation between different-mode and same-mode switches further allows a precise characterization of the conditions under which reset is beneficial versus unnecessary.

**Figure 4:**
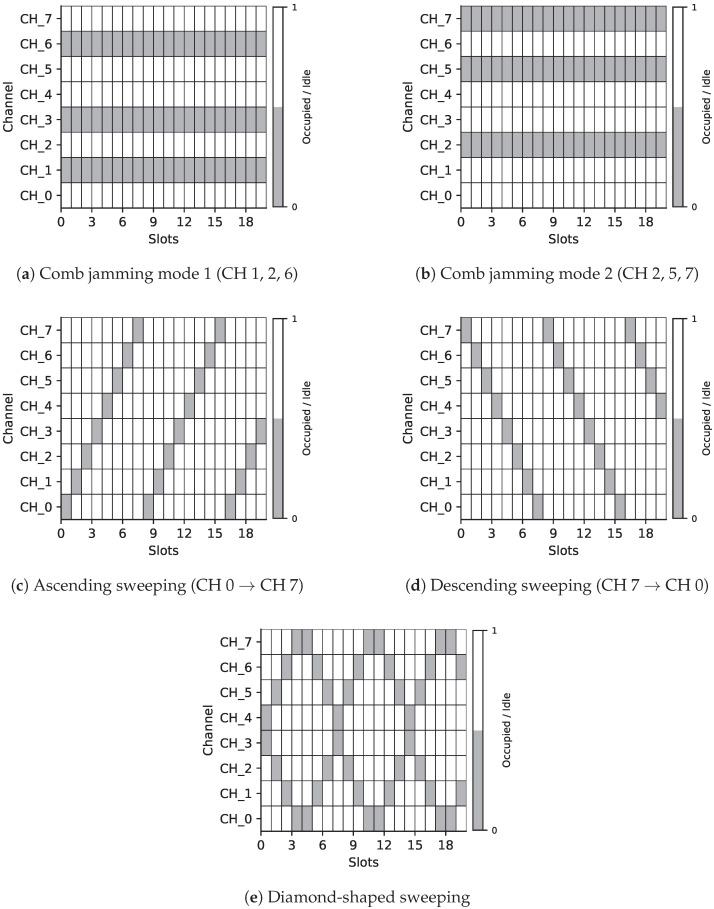
Basic jamming modes. White = idle, dark = jammed.

Figure 4 shows the five basic jamming modes used in the experiments.

Six schemes are compared: DQN-ESN Reset, DQN-ESN NoSet, DQN-LSTM Reset, DQN-LSTM NoSet, ESN-BEDQN Reset, and ESN-BEDQN NoSet. Reset schemes use the known change time to trigger reset, clearing the experience buffer and resetting the network state; NoSet schemes continue without reset. DQN-ESN and DQN-LSTM use ε-greedy (ε≈0.01 at the change point); ESN-BEDQN uses Bootstrap ensemble (five heads).

**Figure 5:**
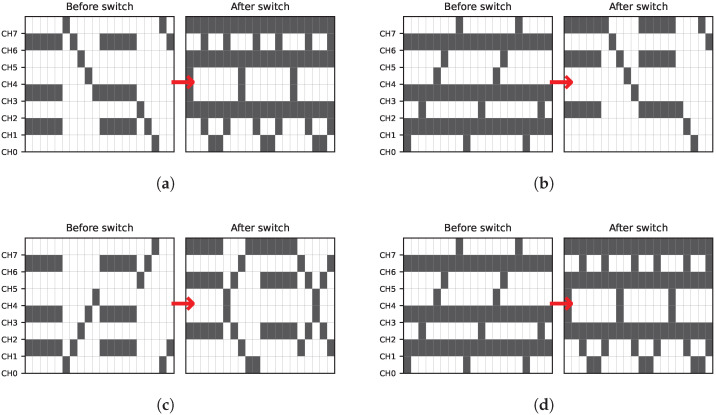
Spectrum occupancy grids for the four scenarios. Each subfigure shows 20 slots before and 20 slots after the switch, separated by the red dashed line. White = idle, dark = jammed. (**a**) Scenario 1: Alternating → Superposition. (**b**) Scenario 2: Superposition → Alternating. (**c**) Scenario 3: Alternating A → Alternating B. (**d**) Scenario 4: Superposition A → Superposition B.

**Figure 6:**
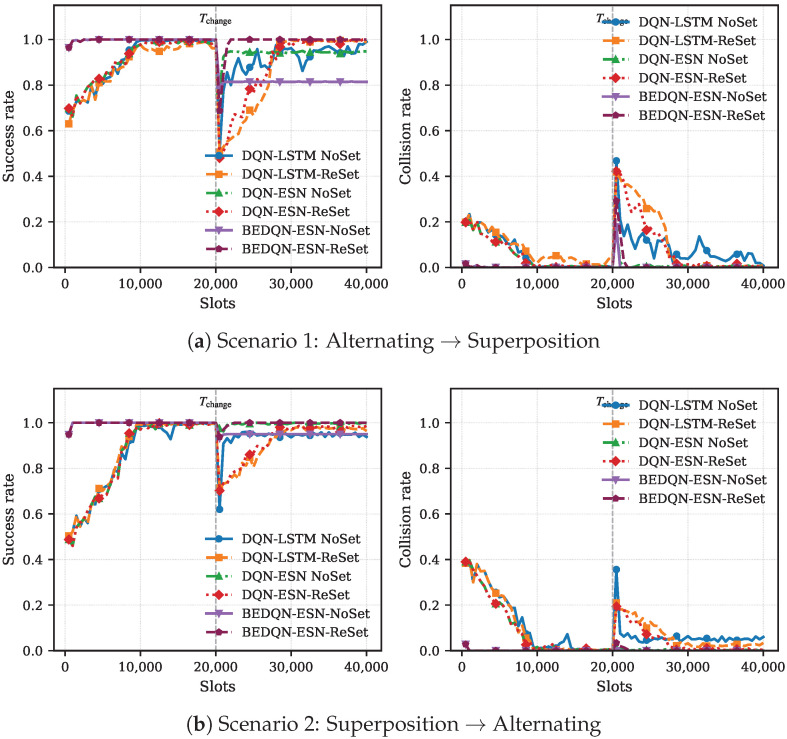

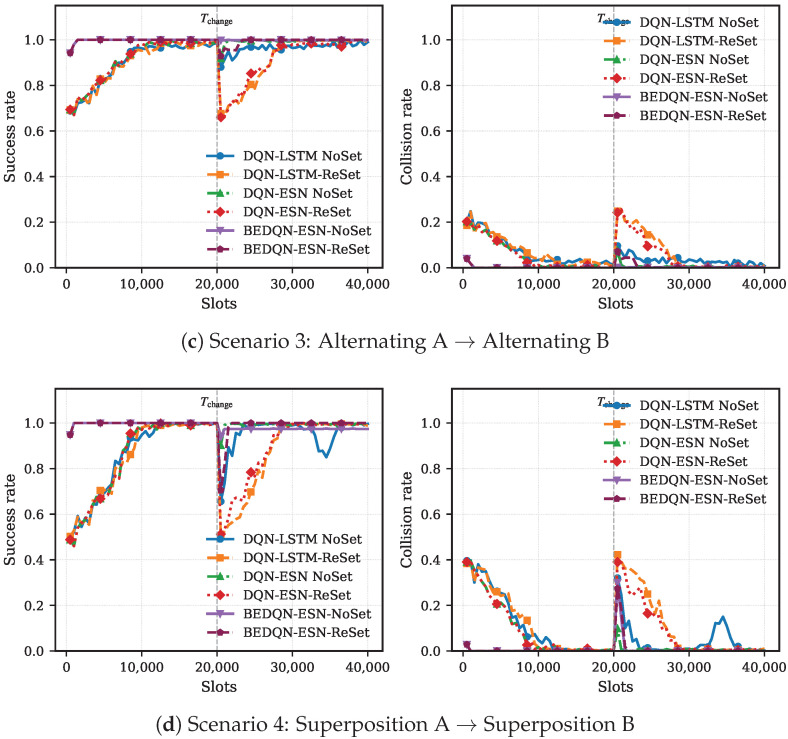
Success access rate and collision rate curves under the four scenarios. Solid lines = Reset, dashed/dotted lines = NoSet. The gray dashed line marks the change point. The results correspond to representative runs and illustrate the typical performance trends.

Figure 5 presents the spectrum occupancy grids for the four scenarios. Figure 6 shows the success access rate and collision rate curves. Table 3 summarizes the recovery steps.

The results exhibit a clear dichotomy. Under different-mode switches (Scenarios 1 and 2), all NoSet schemes failed to recover within the training horizon, while all Reset schemes recovered successfully. ESN-BEDQN Reset achieved the fastest recovery across all four scenarios, approximately 2–6 times faster than DQN-ESN Reset and DQN-LSTM Reset. This demonstrates that when the jamming structure changes fundamentally, old experience cannot transfer and a hard reset is necessary for policy rebuilding. The Bootstrap ensemble provides higher exploration efficiency than ε-greedy after reset, enabling faster policy convergence. During the stationary period before the switch, ESN-BEDQN achieves a steady-state success rate comparable to DQN-ESN, indicating that the random head selection in the Bootstrap ensemble does not introduce excessive exploration noise in stable environments.

Under same-mode switches (Scenarios 3 and 4), a different pattern emerges. All NoSet schemes were able to recover autonomously, demonstrating that temporal memory alone suffices when the jamming structure remains similar. Notably, ESN-BEDQN NoSet recovered in fewer than 100 steps in Scenario 3 and within approximately 1000 steps in Scenario 4, substantially faster than its ε-greedy counterparts. DQN-ESN NoSet and DQN-LSTM NoSet also recovered without reset, but DQN-LSTM NoSet required significantly more steps in Scenario 3. For ε-greedy schemes, the NoSet versions often recovered faster than their Reset counterparts in Scenarios 3 and 4 (Table 3), because after reset, the exhausted exploration capability of ε-greedy slowed convergence, whereas NoSet versions could directly fine-tune the already learned similar policy. In contrast, ESN-BEDQN maintained a positive reset benefit even under same-mode switches, as the Bootstrap ensemble retains exploration capability regardless of the reset timing.

Table 4 compares the reset benefit ratio. Under different-mode switches, all NoSet schemes failed to recover, confirming the indispensability of reset when the jamming structure changes fundamentally. Under same-mode switches, the benefit is marginal or even negative for ε-greedy schemes, while ESN-BEDQN maintains a clear advantage with reset. ESN-BEDQN achieves the best recovery speed among all Reset schemes across all four scenarios.

In summary, the experiments in this section reveal a key insight for temporal network-based dynamic spectrum access: when the jamming modes before and after a switch share a similar structure, the agent can smoothly adapt via temporal memory without reset; when the jamming structure undergoes a fundamental change, old experience becomes invalid and a hard reset is necessary for policy rebuilding. ESN-BEDQN, combining the lightweight temporal memory of ESN, the high exploration efficiency of the Bootstrap ensemble, and the reset mechanism targeting different-mode switches, achieves optimal adaptation performance across all four scenarios.

### 4.4. Validation of Online Detection Effectiveness

The results of Section 4.3 demonstrate that hard reset is the necessary foundation for policy rebuilding under different jamming mode switches. However, the reset schemes in that section all relied on the known change time to trigger reset. In practical deployment, the SU cannot know when the environment changes and must autonomously determine the reset timing through online detection. This section verifies whether Pred-Reset can replace the ideal signal with an acceptable delay.

The experimental setup is identical to Section 4.3, with each of the four scenarios tested. The complete ESN-BEDQN (Bootstrap ensemble, five heads) is used, and two triggering methods are compared:

- Perf-Reset (perfect reset): Reset is triggered immediately at the known change time.
- Pred-Reset (prediction-based reset): The symmetric KL divergence $D_{t}$ between the current idle-ratio vector and that of 30 steps ago is computed in real time. The exponentially weighted moving mean $\mu_{D}$ and standard deviation $\sigma_{D}$ of $D_{t}$ are maintained (update rate α=0.01). Reset is triggered when $D_{t}>\mu_{D}+\beta \cdot \sigma_{D}$, where the sensitivity coefficient β=2.5. After triggering, a cooldown period of 500 steps is entered.

**Figure 7:**
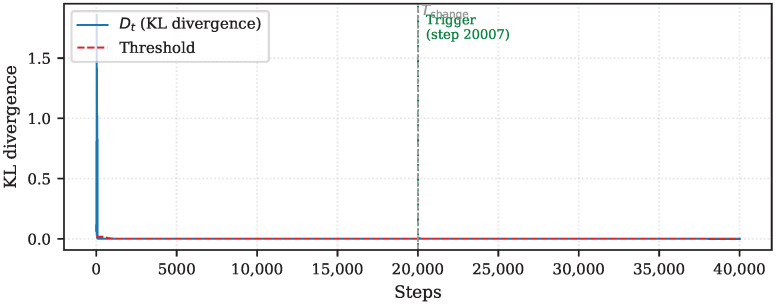
KL divergence $D_{t}$ and dynamic threshold under Scenario 1. The green dashed line marks the Pred-Reset trigger step. The gray dashed line marks the environmental change.

Figure 7 takes Scenario 1 as an example to show the $D_{t}$ curve and dynamic threshold over the entire training process. During the initial start-up phase, due to unstable statistics, $D_{t}$ exhibits a transient spike that triggers a false reset. As samples accumulate, the threshold rapidly converges to a stable range. After the environmental change, $D_{t}$ rises sharply and exceeds the threshold at step 20,002, triggering reset with a delay of only two slots compared to the ideal signal.

**Figure 8:**
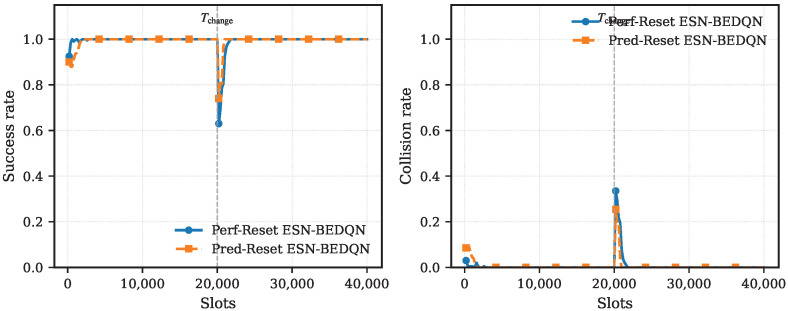
Success access rate and collision rate curves of ESN-BEDQN under Perf-Reset and Pred-Reset. The gray dashed line marks the environmental change.

Figure 8 compares the success access rate and collision rate curves of ESN-BEDQN under Perf-Reset and Pred-Reset. Despite one false trigger during the cold-start period, the recovery curves of the two methods almost completely overlap after the change, with no statistical difference in steady-state performance. Table 5 lists the recovery steps: Pred-Reset achieves a recovery step count comparable to Perf-Reset, with only a few steps of detection delay, representing negligible performance loss in exchange for fully autonomous change adaptation capability.

To further verify the performance of Pred-Reset during the stable period, Table 6 presents the trigger delay and false trigger count around the change moment across all four scenarios (excluding the cold-start period). In Scenario 1 and Scenario 2 (different-mode switches), Pred-Reset triggers reset within a few steps after the change, with zero false triggers during the stable period. In Scenario 3 and Scenario 4 (same-mode switches), Pred-Reset does not trigger any reset. This result is highly consistent with the finding of Section 4.3—no reset is needed under same-mode switches, and Pred-Reset correctly identifies the magnitude of environmental change, avoiding unnecessary reset operations.

The sensitivity coefficient β controls the trade-off between detection delay and false-alarm rate. A larger β reduces false triggers during stationary periods but increases detection delay; a smaller β triggers faster but risks false positives. The cooldown period prevents consecutive re-triggers when $D_{t}$ remains temporarily elevated after the initial change. The chosen values (β=2.5, cooldown = 500 steps) balance these factors across the tested scenarios. In summary, Pred-Reset makes correct decisions across all four scenarios: it triggers reset with a minimal delay of only a few steps under different-mode switches and remains silent under same-mode switches. This detection operates directly on spectrum sensing statistics with extremely low computational overhead, and is highly consistent with the lightweight design philosophy of ESN-BEDQN.

## 5. Conclusions

This paper proposed ESN-BEDQN, a Bootstrap ensemble deep reinforcement learning algorithm for dynamic spectrum access under abrupt jamming pattern changes. The algorithm integrates an echo state network with idle-ratio change detection to address the challenges posed by dynamic jamming pattern switches. The algorithm employs an ESN as the Q-network backbone, exploiting the inherent echo state property of the reservoir to capture temporal regularities in jamming patterns, with only the readout weights requiring training and the online computational cost being substantially lower than that of an LSTM of comparable scale. A Bootstrap ensemble method is adopted to maintain multiple diversified readout heads, naturally sustaining exploration through random head selection and providing higher exploration efficiency than conventional ε-greedy strategies after reset. Furthermore, an adaptive reset mechanism termed Pred-Reset, based on KL divergence change detection of channel idle ratios, immediately clears the experience buffer and resets the reservoir state upon detecting an environmental change, enabling the agent to rapidly adapt to the new environment.

Simulation experiments sequentially verified the three core capabilities of the algorithm. Under static periodic jamming, DQN-ESN achieved a near-zero collision rate, whereas DQN-MLP could only degenerate into a memoryless policy, directly confirming the indispensability of temporal memory for dynamic spectrum access. In time-varying scenarios with two jammers acting jointly, the experiments revealed a key insight for temporal network-based DSA: when the jamming modes before and after a switch share a similar structure, the agent can smoothly adapt via temporal memory without reset; when the jamming structure undergoes a fundamental change, old experience becomes invalid and hard reset is necessary for policy rebuilding. Under different-mode switches, ESN-BEDQN Reset recovered substantially faster than the ε-greedy Reset schemes, while all NoSet schemes failed to recover, confirming the indispensability of reset when the jamming structure changes fundamentally. Online detection experiments demonstrated that Pred-Reset makes correct decisions across all four scenarios—triggering reset with a minimal delay under different-mode switches and remaining silent under same-mode switches where temporal memory alone suffices for adaptation.

Although this work focuses on a simplified single-SU scenario, the proposed ESN-BEDQN framework is inherently extensible to more general CRN settings. The primary challenge addressed in this work—abrupt and unpredictable jamming pattern changes—persists, and often intensifies, in more complex scenarios such as multi-SU systems. From a structural perspective, the proposed framework can be naturally adapted. In multi-SU scenarios, each user can maintain an individual ESN encoder for local observation modeling, while the Pred-Reset mechanism can be extended to a distributed or consensus-based change detection scheme. Additional factors such as user mobility and complex interference primarily affect the state transition dynamics, while the core adaptation loop of ESN-BEDQN remains applicable. Additionally, the current study assumes ideal spectrum sensing and neglects receiver noise. In practice, sensing imperfections such as missed detections and false alarms would affect the ACK-derived idle-ratio statistics on which Pred-Reset relies. Extending the framework to handle non-ideal sensing is another important direction for future work. However, more challenging adversarial settings, such as intelligent or tracking jammers that actively react to SU behavior, may introduce stronger coupling between agents and environments, making jamming pattern changes more complex and harder to detect. In such cases, the effectiveness of the current Pred-Reset mechanism may be affected and requires further investigation. Experimental validation in these more complex and adversarial scenarios is left for future work.

## Figures and Tables

**Table 1:** Key hyperparameters.

Parameter	Value
Number of channels *K*	4 (Section 4.2)/8 (Section 4.3 and Section 4.4)
Reservoir size $N_{\mathrm{res}}$	64
Spectral radius ρ	0.8
Readout learning rate	0.01
Discount factor γ	0.9
Replay buffer capacity *M*	290
Mini-batch size	32
Target network update interval *C*	10
Bootstrap ensemble heads $N_{\mathrm{ensemble}}$	5
Bernoulli mask probability $p_{\mathrm{mask}}$	0.6
ε decay range	1.0 → 0.01
ε decay steps	10,000
Idle-ratio window length *W*	200
Pred-Reset comparison interval	30
Pred-Reset update rate α	0.01
Pred-Reset sensitivity β	2.5
Pred-Reset cooldown period	500 steps
Intrinsic reward weight λ	0.1
Optimizer	SGD (all algorithms)

**Table 2:** Steady-state average collision rate (static jamming).

Algorithm	T=3	T=5
DQN-ESN	≈0%	≈0%
DQN-MLP	33.3%	20.0%

**Table 3:** Policy recovery steps under the four scenarios.

Scheme	Sc. 1	Sc. 2	Sc. 3	Sc. 4
ESN-BEDQN Reset	2185±96	2028±87	3612±156	2216±108
DQN-ESN Reset	13,240 ± 820	9735±690	16,980 ± 1380	8725±760
DQN-LSTM Reset	9870±730	9655±810	8860±720	8750±680
ESN-BEDQN NoSet	–	–	86±12	1045±95
DQN-ESN NoSet	–	–	1655±145	935±110
DQN-LSTM NoSet	–	–	19,180 ± 1620	5180±460

Recovery steps are defined as the number of steps required to regain 90% of the steady-state performance in the new environment. “–” indicates that the scheme failed to recover within the total training steps.

**Table 4:** Reset benefit ratio (recovery steps NoSet/Reset).

Scheme	Sc. 1	Sc. 2	Sc. 3	Sc. 4
ESN-BEDQN	–	–	<0.03×	0.45×
DQN-ESN	–	–	0.10×	0.11×
DQN-LSTM	–	–	2.17×	0.60×

“–” indicates that the NoSet version failed to recover within the total training steps. A ratio >1 indicates that reset accelerates recovery; <1 indicates that the NoSet version recovers faster.

**Table 5:** Perf-Reset vs. Pred-Reset (Scenario 1).

Trigger Method	Recovery Steps	Detection Delay	False Triggers
Perf-Reset	512±44	0	0
Pred-Reset	537±51	4±1	1 (cold-start)

**Table 6:** Pred-Reset performance across four scenarios (stable period).

Scenario	Trigger Delay	False Triggers
Scenario 1 (Different-mode)	4±1	1 (cold-start)
Scenario 2 (Different-mode)	7±1	1 (cold-start)
Scenario 3 (Same-mode)	Not triggered	1 (cold-start)
Scenario 4 (Same-mode)	Not triggered	1 (cold-start)

## Data Availability

The original contributions presented in this study are included in the article. Further inquiries can be directed to the corresponding author.

## References

[1] Tan X., Zhou L., Wang H., Sun Y., Zhao H., Seet B.C., Wei J., Leung V.C. (2022). Cooperative multi-agent reinforcement-learning-based distributed dynamic spectrum access in cognitive radio networks. IEEE Internet Things J..

[2] Alsaedi W.K., Ahmadi H., Khan Z., Grace D. (2023). Spectrum options and allocations for 6G: A regulatory and standardization review. IEEE Open J. Commun. Soc..

[3] Khasawneh M., Azab A., Alrabaee S., Sakkal H., Bakhit H.H. (2023). Convergence of IoT and cognitive radio networks: A survey of applications, techniques, and challenges. IEEE Access.

[4] Kaur A., Kumar K. (2022). A comprehensive survey on machine learning approaches for dynamic spectrum access in cognitive radio networks. J. Exp. Theor. Artif. Intell..

[5] Jia L., Qi N., Su Z., Chu F., Fang S., Wong K.K., Chae C.B. (2024). Game theory and reinforcement learning for anti-jamming defense in wireless communications: Current research, challenges, and solutions. IEEE Commun. Surv. Tutor..

[6] Dai Y., Lyu L., Cheng N., Sheng M., Liu J., Wang X., Cui S., Cai L., Shen X. (2024). A survey of graph-based resource management in wireless networks—Part I: Optimization approaches. IEEE Trans. Cogn. Commun. Netw..

[7] Janu D., Singh K., Kumar S. (2022). Machine learning for cooperative spectrum sensing and sharing: A survey. Trans. Emerg. Telecommun. Technol..

[8] Li Y., Zhang W., Wang C.X., Sun J., Liu Y. (2020). Deep reinforcement learning for dynamic spectrum sensing and aggregation in multi-channel wireless networks. IEEE Trans. Cogn. Commun. Netw..

[9] Bokobza Y., Dabora R., Cohen K. (2023). Deep reinforcement learning for simultaneous sensing and channel access in cognitive networks. IEEE Trans. Wirel. Commun..

[10] He M., Jin M., Guo Q., Xu W. (2023). Listen-after-collision mechanism for dynamic spectrum access using deep Q-network with an improved Thompson sampling algorithm. IEEE Internet Things J..

[11] Xu Y., Yu J., Buehrer R.M. (2020). The application of deep reinforcement learning to distributed spectrum access in dynamic heterogeneous environments with partial observations. IEEE Trans. Wirel. Commun..

[12] Zhong C., Lu Z., Gursoy M.C., Velipasalar S. (2019). A deep actor-critic reinforcement learning framework for dynamic multichannel access. IEEE Trans. Cogn. Commun. Netw..

[13] Tang C., Chen Y., Chen G., Du L., Liu H. (2024). A dynamic and collaborative spectrum sharing strategy based on multi-agent DRL in satellite-terrestrial converged networks. IEEE Trans. Veh. Technol..

[14] Ngo Q.T., Jayawickrama B.A., He Y., Dutkiewicz E. (2023). Multi-agent DRL-based RIS-assisted spectrum sensing in cognitive satellite–terrestrial networks. IEEE Wirel. Commun. Lett..

[15] Zhang W., Ding W., Wang C.X., Chen Y., de Abreu G.T.F., Liu Z., Sun J., Han Z. (2025). Dynamic spectrum aggregation and access for rescue cognitive networks using multi-agent actor-critic reinforcement learning. IEEE Trans. Cogn. Commun. Netw..

[16] Liu X., Li X., Zheng K., Liu J. (2024). AoI minimization of ambient backscatter-assisted EH-CRN with cooperative spectrum sensing. Comput. Netw..

[17] Dong X., You Z., Liu X., Guo Y., Shen Y., Gong Y. (2023). Federated and online dynamic spectrum access for mobile secondary users. IEEE Trans. Wirel. Commun..

[18] Jameel A.S.M.M., Mohamed A.P., Zhang X., El Gamal A. (2021). Deep learning for frame error prediction using a DARPA spectrum collaboration challenge (SC2) dataset. IEEE Netw. Lett..

[19] Bowyer C., Greene D., Ward T., Menendez M., Shea J., Wong T. (2019). Reinforcement learning for mixed cooperative/competitive dynamic spectrum access. Proceedings of the 2019 IEEE International Symposium on Dynamic Spectrum Access Networks (DySPAN).

[20] Dulac-Arnold G., Levine N., Mankowitz D.J., Li J., Paduraru C., Gowal S., Hester T. (2020). An empirical investigation of the challenges of real-world reinforcement learning. arXiv.

[21] Pascanu R., Mikolov T., Bengio Y. (2012). On the difficulty of training Recurrent Neural Networks. arXiv.

[22] Wang S., Liu H., Gomes P.H., Krishnamachari B. (2018). Deep reinforcement learning for dynamic multichannel access in wireless networks. IEEE Trans. Cogn. Commun. Netw..

[23] Sheng H., Zhou W., Zheng J., Zhao Y., Ma W. (2023). Transfer reinforcement learning for dynamic spectrum environment. IEEE Trans. Wirel. Commun..

[24] Albinsaid H., Singh K., Biswas S., Li C.P. (2021). Multi-agent reinforcement learning-based distributed dynamic spectrum access. IEEE Trans. Cogn. Commun. Netw..

[25] Jaeger H. (2001). The “Echo State” Approach to Analysing and Training Recurrent Neural Networks-with an Erratum Note.

[26] Chang H.H., Mohammadi N., Safavinejad R., Yi Y., Liu L. (2024). Dyna-ESN: Efficient deep reinforcement learning for partially observable dynamic spectrum access. IEEE Trans. Wirel. Commun..

[27] Safavinejad R., Chang H.H., Liu L. (2024). Deep reinforcement learning for dynamic spectrum access: Convergence analysis and system design. IEEE Trans. Wirel. Commun..

[28] Osband I., Blundell C., Pritzel A., Van Roy B. (2016). Deep exploration via bootstrapped DQN. Advances in Neural Information Processing Systems.

[29] Li L., Liu L., Bai J., Chang H.H., Chen H., Ashdown J.D., Zhang J., Yi Y. (2020). Accelerating model-free reinforcement learning with imperfect model knowledge in dynamic spectrum access. IEEE Internet Things J..

